# Predicting the Optical Properties of Gold Nanoclusters Using Machine Learning Approach

**DOI:** 10.1021/acsomega.5c06771

**Published:** 2025-10-17

**Authors:** Geraldine Sánchez-Dueñez, Wladimiro Diaz-Villanueva, Jorge Escorihuela, Laura Francés-Soriano, Julia Pérez-Prieto

**Affiliations:** † Institut de Ciència Molecular (ICMol), 16781 Universitat de València, C/Catedrático José Beltrán 2, 46980 Paterna, Spain; ‡ Institute for Integrative Systems Biology (I2SysBio), Universitat de València/CSIC, C/Catedràtic Agustín Escardino Benlloch, 46980 Valencia, Spain; § Departamento de Química Orgánica, Facultad de Farmacia y Ciencias de la Alimentación, Universitat de València, Avda. Vicente Andrés Estellés s/n, 46100 Burjassot, Spain; ∥ Grupo de Procesos de Oxidación Avanzada, Departamento de Ingeniería Textil y Papelera, 16774 Universitat Politècnica de València, Campus d’Alcoi, 03801 Alcoi, Spain

## Abstract

The synthesis of gold nanoclusters (AuNC) is strongly influenced by various reaction conditions, and their optical properties are determined by factors such as the nature of the ligand and the measuring solvent, among others. To improve the efficiency of the synthesis of metallic gold nanoclusters with the desired functionality, the application of machine learning techniques is a smart choice. In this study, a model based on the GXBoost algorithm is proposed to predict the maximum emission wavelength of the AuNC emission from a database that includes more than 200 scientific articles. The validation of the model was carried out through the comparison of prediction versus experimental data (not included in the model) and the training and validation data. The model showed a percentage error of 1.7, 1.6, and 4.9%, respectively, indicating a reasonable return. Additionally, an independent regression was performed when the ligand was a thiolated compound different from GSH, obtaining a training and test error percentage of 0.01 and 3%, respectively. In addition, critical variables affecting the optical properties of nanoclusters were explored, and techniques such as One-Hot Encoding were used to prepare the data. Finally, this work not only underscores the relevance of AuNCs in modern science highlighted but also demonstrates the potential of machine learning as a predicting tool and design of materials in nanoscience, contributing to the optimization of their properties for future applications.

## Graphical abstract

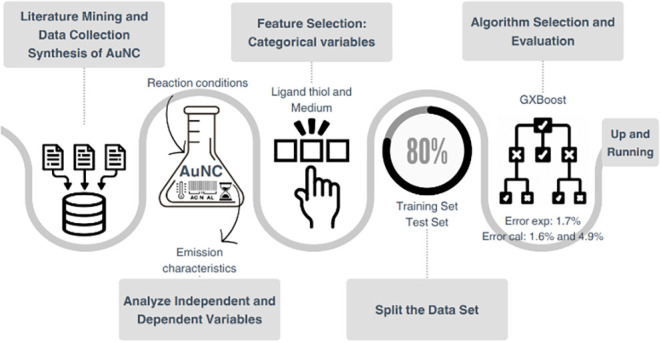

## Introduction

The ongoing quest for new tools and methodologies to predict and control the physicochemical properties of nanomaterials has become a central topic in the field of nanoscience and nanotechnology.^1^ Among the wide diversity of nanostructured materials, gold nanoclusters (AuNCs) have gained substantial attention due to their unique quantum confinement effects, discrete energy levels, and strong photoluminescence, particularly in the near-infrared (NIR) region.^2,3^ These properties, which emerge when the size of gold particles is reduced to the sub-2 nm scale, make AuNCs highly promising for a wide range of applications, such as biomedical imaging, drug delivery, catalysis, sensing, and photonics.^4–9^

A critical aspect for exploiting the full potential of AuNCs lies in understanding and predicting their optical properties, with particular emphasis on their emission wavelength.^10–12^ Thus, the ability to tailor the maximum emission wavelength of AuNCs during synthesis has become a key objective in the rational design and development of next-generation AuNC-based nanomaterials.^13,14^ This capability not only enables the fabrication of tailored nanoclusters for specific functions but also paves the way for innovative applications in technological fields, including bioimaging, sensing, and optoelectronics.

The emissive properties of AuNCs are significantly affected by a variety of parameters associated with their synthesis.^15^ These factors include the core size of the cluster, crystal structure, the nature and density of surface ligands, stabilizing agents, the reaction temperature, pH conditions, and time.^10,16–20^ Therefore, careful control and optimization of synthesis conditions not only enables the fabrication of tailored nanoclusters for specific functions but also paves the way for innovative applications in technological fields, including bioimaging, sensing, and optoelectronics.^21,22^ Thiol-containing ligands have proven to be especially effective in stabilizing AuNCs, preventing aggregation, and tuning their photoluminescence behavior by modulating surface states and charge transfer processes.^23,24^ Despite these advances, the multifactorial nature of these dependencies introduces significant challenges in rationally predicting the optical outcomes of a given synthetic route. Consequently, traditional empirical approaches (generally based on trial-and-error experimentation involving the selection of reaction conditions, ligands, or Au precursors, followed by multistep chemical synthesis, purification, and characterization) are laborious and time-consuming, hampering the efficiency and scalability of developing novel tailored AuNC-based materials with desired optical features.^25,26^

In this context, machine learning offers powerful capabilities such as nonlinear mapping, unsupervised learning, and precriminal computational capacity, allowing the modeling of the complex relationships governing AuNC emission properties. As a result, accurately defining and predicting the maximum emission wavelength of AuNCs through machine learning represents a significant step forward in terms of precise control over their optical properties during synthesis. Next, the main learnings from machine learning will be mentioned, specifically the characterization of AuNC nanoclusters, the most influential variable within the prediction model, the result of the algorithm, testing with experimental data not used within the data set, but synthesized, and the error when using the data uploaded to the model.^27^

Machine learning constitutes an innovative approach to address these challenges.^27,28^ Machine learning algorithms, particularly those capable of capturing complex, nonlinear relationships, have shown remarkable success in the field of materials science applications, including property prediction, synthesis optimization, and structure–function analysis.^29^ Ensemble learning methods such as Extreme Gradient Boosting (XGBoost) have emerged as powerful tools for regression tasks due to their ability to capture complex, nonlinear relationships and improve prediction accuracy by iteratively combining multiple weak learners. Originally introduced by Chen and Guestrin,^30^ XGBoost offers potential advantages including robustness to overfitting through built-in regularization, efficient handling of missing data, and scalability for large data sets. These capabilities make XGBoost particularly well-suited for materials science applications, where data sets often involve numerous interdependent variables and noise, incomplete data.^31,32^ Its widespread success in both academic and industrial settings underscores its versatility and effectiveness in a wide range of predictive modeling problems.^33–35^

Recent studies have shown that machine learning techniques are highly effective in predicting a wide range of properties of nanomaterials.^11,36–40^ By using large data sets and advanced algorithms, these approaches can associate complex relationships between structure and properties, enabling more accurate and efficient characterization, optimization, and even the discovery of novel nanomaterials with tailored properties.^41,42^ Artificial neural network models were employed for the accurate prediction of the polarizability of AuNCs using high-order invariant descriptors based on spherical harmonics.^43^ The stability of the AuNCs with different ligands and metal cores has been predicted using a convolution neural network model, allowing a fast screening of multiple AuNC structures and substantially reducing the need for multiple trials.^44^ Moreover, machine learning-calculated interatomic potentials have allowed the prediction of complex interactions in gold–thiolated nanoclusters, capturing dynamic behaviors as well as intercluster processes, i.e., isomerization, coalescence, and ring formation.^45^ More interestingly, machine learning methods also hold promise for the development of cost-effective, versatile, and highly accurate sensing systems. Noreldeen et al. developed a machine learning sensor using random forest and linear discriminant analysis and fluorescence data from gold nanoclusters to detect and distinguish heavy metal ions and anions.^46^ Despite the fast development of predictive models for AuNCs, there is no model for predicting the emission wavelength based on synthetic parameters and structural features unique to AuNCs.

In this work, we propose a predictive model based on The GXBoost Regressor (Adaptative Boosting Regressor) to estimate the maximum emission wavelength of thiol-stabilized AuNCs from key synthetic parameters such as cluster size, pH, synthesis temperature, and reaction time. Trained on a curated data set compiled from over 200 scientific articles, the model enables accurate, data-driven prediction of photoluminescent properties, offering a valuable prediction tool for guiding experimental design and reducing reliance on trial-and-error methods. To validate the model, we synthesized AuNCs using glutathione as a thiolated ligand, and the experimental emission wavelength was compared with model predictions, demonstrating strong alignment and practical utility.

## Results and Discussion

Although experimental and computational methods can be used to obtain emission data for AuNCs, these approaches are often time-consuming, particularly when they are applied to large sample sets. In particular, computational approaches, such as quantum mechanical calculations and molecular simulations, can demand substantial computational power and extended processing times when applied to complex and large systems. As an alternative approach, utilizing reliable data sets compiled from previously published scientific literature offers a more practical and efficient strategy. Using this existing data enables the development of an ML model to investigate and optimize the emission properties of AuNCs.^47^

In the context of nanoparticle synthesis, the relationship between synthesis conditions and the resulting optical properties is inherently complex, making it well-suited for ML-based regression modeling.^31^ Among various algorithms, the XGBoost model was selected due to its high predictivity accuracy, low computational cost, and ease of implementation. XGBoost enhances performance by combining multiple weak learners, effectively reducing both bias and variance. Additionally, XGBoost does not require data scaling, which clearly simplifies data processing. Its compatibility with decision-tree-based classifiers also lends flexibility and adaptability across diverse data sets.

The predictive performance of the model was influenced by key factors, including the size of the test set, the selection of the kernel function, and the definition of the loss function. Although large data sets generally improve predictive accuracy, they also increase computational costs, processing time, and the risk of overfitting.^48^ Thereby, a moderate data set comprising 207 entries was used in this model. The data were randomly divided into a training set (80%) and a test set (20%), using random sampling to minimize the risk of overtraining. To preserve the integrity of the model evaluation, the test set remained untouched during the training without employing extensive cross-validation. Moreover, an independent experimental sample was selected as an external validation set and completely excluded from model development, further strengthening the reliability of model evaluation.

The target variable was the experimentally determined emission wavelength (in nanometers), considering the set of synthesis variables: particle size (nm), pH, temperature (°C), synthesis time (h), ligand, and measurement solvent as input features. Categorical variables, i.e., ligand and solvent, were encoded using an ordinal encoder, while numerical features were used in their raw form, as XGBoost’s tree-based architecture eliminates the need for normalization or standardization. To assess feature importance, One-Hot Encoding was applied during training, and the relative influence was quantified through the average gain in the tree splits (Table 1).

**Table 1:** Relative Importance of Features Calculated through Their Average Gain Values

Feature	Importance
synthesis time	0.4020
temperature	0.2009
ligand	0.1905
solvent	0.0727
particle size	0.0671
pH	0.0669

As inferred from Table 1, the most influential factors in the model were the synthesis time (0.4020) and temperature (0.2009), followed by ligand type (0.1905). In contrast, particle size and pH exhibited lower impact on the predictive model, likely due to their limited variability in the data set (*e.g.*, particle size: 1.6–2.2 nm; >80% of pH values were neutral). Figure S1 shows the relationship between the synthesis variables and the emission wavelength, demonstrating that variables such as AuNC size and pH of the cluster remain constant at their average value, forming a “cloud” around a single value. This is consistent with common practice in AuNC synthesis, where particles typically have an average size of *ca.* 2 nm and the synthesis pH is typically near 7. One-Hot Encoding helped to eliminate any implicit ranking between categorical values, reducing the risk of introducing spurious relationships into the model.^49^

XGBoost operates by iteratively adjusting the weights of the training instances. In each iteration, a weak classifier (typically a decision tree) is trained on the weighted data set. Misclassified instances were assigned higher weights in subsequent rounds, allowing the model to progressively focus on harder-to-predict samples. This boosting process was repeated multiple times, with each new tree attempting to correct the errors of the previous ensemble. Finally, the results from all classifiers were combined to generate the model’s final prediction. This approach enables XGBoost to progressively improve its performance and effectively handle complex data sets.

The model demonstrated a solid performance in the test set, with relative residuals ranging from −30% to +16%, an acceptable spread given the nonlinear and multiparametric nature of the problem. The distribution of normalized residuals was examined using a quantile–quantile plot (Q–Q plot, Figure 1), revealing that the residuals follow a near-normal distribution.

**Figure 1:**
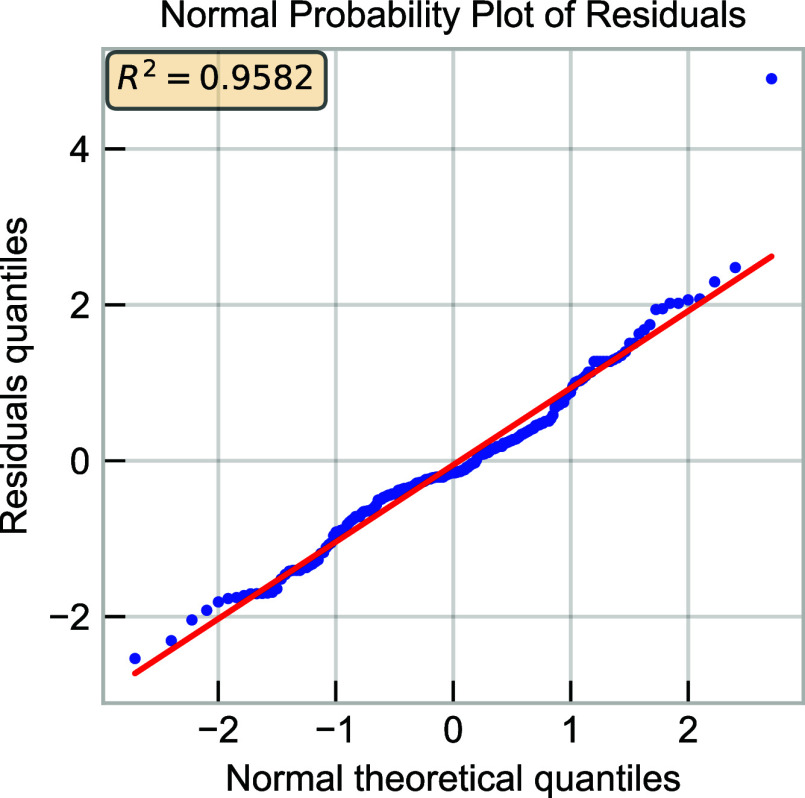
Normal Q–Q plot of standardized residuals. Residuals (blue dot) compared to the expected normal distribution (red line).

As shown in Figure 1, the residuals align closely with the expected normal distribution, confirming that they are approximately normally distributed. Minor deviations at the tails suggested the presence of some outliers or slight heavy-tailed behavior, but without statistical significance. The model achieved a coefficient of determination of *R* ^2^ = 0.9582, indicating a high degree of fit and a strong predictive capability. Overall, the assumption of residual normality was reasonably met, further validating the model’s reliability in predicting the emission properties of AuNCs.

### Model Evaluation

To evaluate the performance of the developed ML model in predicting the maximum emission wavelength of thiolate-protected AuNCs, an independent experimental sample, entirely excluded from both the training and testing data sets, was used as a validation point. To this purpose, glutathione-stabilized AuNCs (AuNC@GSHs) were synthesized following a previously described protocol.^50^ Briefly, a mixture of an aqueous solution of trihydrate gold­(III) chloride and l-glutathione was stirred at room temperature (1.5 h). Then, the reaction mixture was heated at 70 °C for 24 h. After cooling down to room temperature, the AuNC@GSHs were purified by using acetonitrile and several centrifugation steps.

The resulting AuNC@GSH dispersion appeared as a pale-yellow transparent solution under daylight with an orange fluorescence under a UV lamp (Figure S2). Figure 2 shows the UV–Vis absorption spectrum of the AuNC@GSH where a broad absorption band centered at 400 nm with a shoulder around 350 nm was observed. Concerning the emission properties, upon excitation at 400 nm, an emission band with a maximum at 635 nm was observed.

**Figure 2:**
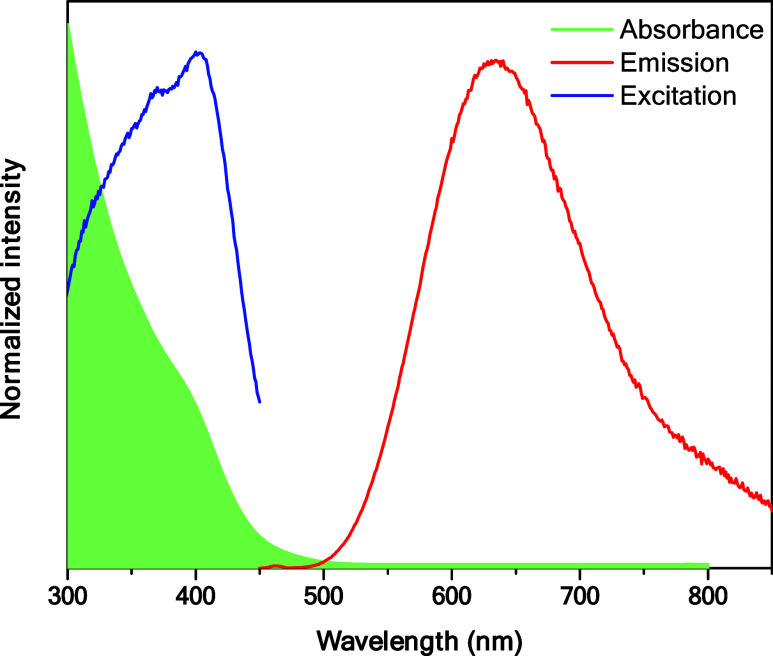
Absorption (green zone), excitation (λ_em_ = 635), and emission (λ_exc_ = 400 nm) spectra of AuNC@GSH in water.

Once AuNC@GSH was optically characterized, a test was carried out using some of the known synthesis data within the model to verify the effectiveness of the prediction. The model predicted a value of 624.5 nm for the maximum emission wavelength (against 635 nm for the observed experimental wavelength), resulting in an absolute error of 10.5 nm and relative error of 1.7%. This low prediction error observed for a previously unseen sample suggested that the model generalizes reasonably well to new data within the experimental domain considered.

Figure 3 displays the predicted values plotted against the actual emission wavelengths across the training, test, and validation subsets. A least-squares regression line is included, yielding an *R* ^2^ of 0.7171. Most predicted values were around the line, especially in the 500–700 nm range, indicating strong model performance in this region. However, the model trended to underestimate emission wavelengths at the higher end of the spectrum. This is likely due to the limited number of AuNCs in the data set emitted at this region, resulting in greater prediction dispersion and limited representation in this range.

**Figure 3:**
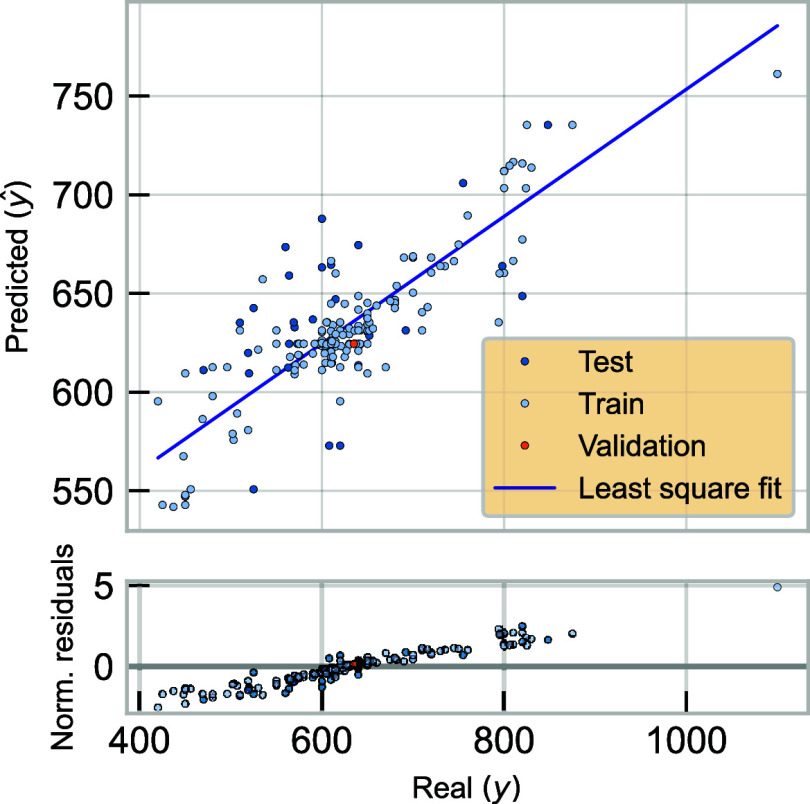
Predicted vs real emission wavelengths for training, testing, and validation sets.

Further analysis was conducted by plotting normalized residuals versus actual values to assess any systematic patterns in the model’s predictions across the range of observed values. Ideally, residuals should appear randomly distributed around zero without a discernible pattern although residuals are generally centered. Figure 3 reveals increasing residual magnitude at higher wavelengths, suggesting heteroskedasticity, where prediction errors grow with increasing target values. This indicates that the model is less effective at capturing variability in the upper range of emission wavelengths. This limitation may be addressed through techniques such as target transformation (*e.g.*, *log­(y)* or Box–Cox­(*y*) transformations), which stabilize variance and improve model robustness at higher values. Additionally, expanding the training set to include more samples with high-wavelength emissions could help the model better learn these patterns.

On the other hand, an independent analysis was conducted for those nanoclusters that used thiol ligands different from GSH (Tables S1 and S2). The results obtained are promising with a training *R* ^2^ of 0.997 (Figure 4), indicating that the model fits exceptionally well to the training data. Moreover, the validation *R* ^2^ of 0.915 (Figure 4) suggests that the model generalizes quite well to unseen data, which is a positive indicator of its predictive capability.

**Figure 4:**
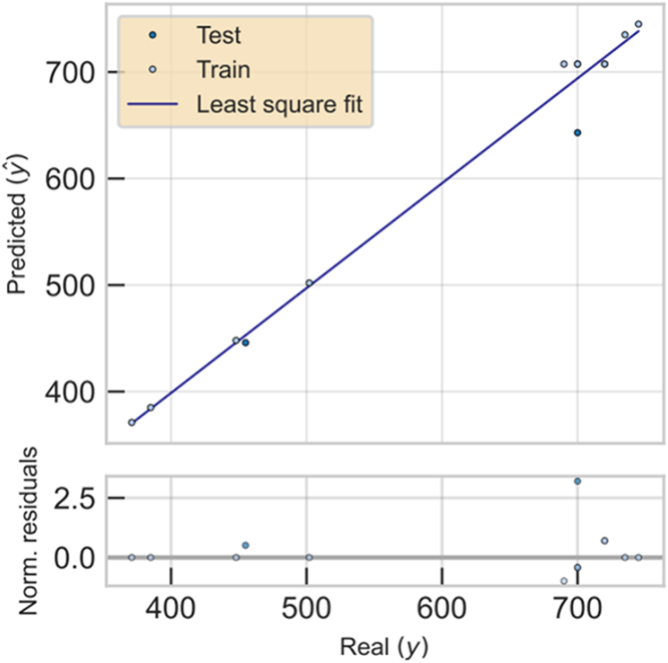
Predicted vs real emission wavelengths for training, testing, and validation sets to thiol ligands.

When thiolated ligands other than GSH were used, One-Hot Encoding was applied in the training set, and the relative influence of variables was quantified through the average gain in tree splits. To better understand the relationship between the ligand type and AuNC optical properties, we also examined studies that employed different thiol ligands and reported the number of gold atoms in the nanocluster structure. A regression model was constructed using a total of 13 data points. A regression model was constructed using a total of 13 data points, with 10 points used for training and 3 reserved for testing model generalization. This analysis enabled the assessment of how variations in ligand type and cluster size influence the emission wavelength of AuNCs. This analysis allowed us to assess how variations in the ligand type and cluster size affect the emission wavelength of AuNCs.

The feature importance results (Table 2) indicate that cluster size and excitation frequency are the dominant factors influencing maximum emission wavelength, showing the strongest correlations with emission behavior. This is because different ligands directly affect the size of nanoclusters, and consequently, in this prediction where different ligands are tested, cluster size becomes a critical variable. These findings suggest that when designing experiments and optimizing AuNC properties with thiol-containing ligands, these two variables should be prioritized. Meanwhile, the synthesis temperature, pH, and synthesis time do not seem to have a significant impact on the optical properties of AuNCs, such as when using GSH as a ligand, indicating that it may be possible to simplify the synthesis process by not requiring such strict control over these variables.

**Table 2:** Relative Importance of Features Calculated through Their Average Gain Values for Thiolate-Capped AuNCs

Feature	Importance
particle size	0.6082
excitation frequency	0.3445
Au atoms	0.0340
solvent	0.0132
temperature	0.0001
synthesis time	0.0000
pH	0.0000

Furthermore, a relative average error of 0.01% can be calculated in the training set, and in the validation set, it is 3% in the emission wavelength predictions (Tables S1 and S2). The model’s capability allows for appropriate generalization supported by a validation *R* ^2^ of 0.915 (Figure 4), suggesting that the predictions are reliable and that the model could be useful in practical applications. However, to further improve the model, it might be advantageous to increase the size of the data set to include more data points from different synthesis conditions and to explore other features that could influence the emission behavior of AuNCs. In this regard, increasing the diversity and volume of the data set can help the model capture a broader range of emission behaviors and improve its generalizability. Furthermore, integrating other potentially relevant features, such as ligand type and reaction temperature, among others, could provide deeper insights into the factors influencing the emission properties of AuNCs and lead to a more accurate and robust predictive model.

In addition to the experimental data, a subset of known synthesis data included in the model training was also tested using the model. This evaluation aimed to validate the model’s ability to reproduce experimentally reported emission wavelengths by comparing the actual values with those predicted by the algorithm. The results of this comparison are listed in Tables S3 and S4, where the real emission values are listed together with the model’s prediction.

Overall, the XGBoost model demonstrated strong predictive performance in low to midrange emission wavelengths, which is where most thiolate-protected AuNCs typically emit. However, the model’s predictive performance declined at higher values, reflecting a decrease in performance compared to the lower and midrange values. One possible contributing factor is the uneven distribution of ligands across the data set. Certain ligands were overrepresented, while others were rare or missing entirely, which could limit the model’s generalization capacity for underrepresented chemical environments. Thus, increasing the diversity of ligands included in the data set would improve the model’s predictive accuracy and robustness across a broader range of gold nanoclusters.

Moreover, the variability in synthesis conditions such as solvent type, temperature, pH, reaction time, and ligand can significantly influence emission properties and introduce additional complexity. Although XGBoost is highly effective at modeling nonlinear relationships within data, the multifactorial nature of nanocluster synthesis introduces additional sources of variability and uncertainty into the predictions.

The quality of the input data also plays a critical role in the prediction accuracy of the model. Some literature-derived data may include incomplete or imprecise experimental details, which affect the quality of the data set used to train the model by introducing noise. This uncertainty limits the model’s ability to accurately learn patterns and can reduce its performance on real-world samples. Therefore, both the quantity and quality of the training data are essential to develop an efficient and accurate prediction model. A more comprehensive and representative data set, covering a broader range of synthesis conditions and emission characteristics, would improve model accuracy and reliability.

As inferred from Tables S3 and S4, the model exhibited a relative average error of 1.6% on the training set and 4.9% on the validation set for the emission wavelength predictions, which is considered acceptable within the typical prediction ranges for this type of model. Although it is possible to continue overtraining the model reaching a relative average error of 2.1% and 7.3%, respectively, we decided to stop the training earlier because the error of the tested value increased from 1.6% to 2.1%, decreasing the efficiency of the model and, in addition, guaranteeing a margin of maneuver.

This low error rate indicates that the prediction model is highly accurate and reliable, suggesting that it is capable of effectively capturing the relationships between the input variables and the optical properties of AuNCs. The model’s ability to maintain such a low error highlights its potential to be used in practical applications, where accuracy in predicting optical properties is critical for the design and optimization of nanoparticles in various disciplines.

## Conclusions

The present study demonstrates the feasibility of using machine learning techniques; specifically, the GXBoost algorithm predicts the maximum emission wavelength of thiolate-protected AuNCs. Through the collection and analysis of data from over 200 scientific publications, a comprehensive data set was developed, integrating key synthesis parameters, such as particle size, ligand nature, medium pH, synthesis temperature, and reaction time. The model exhibited strong predictive power, with a relative error of 1.7% for an independent test sample, 1.6% on the training data, and 4.9% for validation data. Additionally, when the ligand was a thiol different from GSH, a training and test error percentage of 0.01% and 3% was obtained, respectively. These low error margins indicate the model’s capacity to generalize effectively to unseen data, confirming its suitability for guiding experimental design in AuNC synthesis. This approach not only predicts emission properties of AuNCs but also highlights current synthesis challenges, such as variability in conditions and limited control over the optical properties. By quantifying complex relationships between synthesis parameters and emission, machine learning offers a powerful bridge between experimental nanochemistry and data-driven design. This integrative strategy enables the targeted development of AuNCs for sensing, imaging, and photonics, marking a key step toward scalable, customizable nanomaterials with tunable optical properties.

## Methods

### Synthesis of Glutathione-Stabilized Gold Nanoclusters (AuNC@GSHs)

The synthesis of glutathione-protected gold nanoclusters (AuNC@GSHs) was carried out following a modified previously described procedure.^50^ First, 0.2498 g of trihydrate gold­(III) chloride (1.76 mmol) was dissolved in 300 mL of deionized water (DI water) at room temperature in a 500 mL round-bottomed flask. Under continuous stirring, 0.2798 g of l-glutathione (1.22 mmol) was added to the solution. Stirring was continued until the solution turned colorless (*ca.* 1.5 h). The reaction mixture was then heated to 70 °C and pH 7 in an oil bath for 24 h to induce the formation of NCs. After being heated, the flask was allowed to cool to room temperature.

To purify the synthesized AuNC@GSH, 30 mL of acetonitrile was added to 10 mL of the reaction mixture. The resulting solution was mixed thoroughly and centrifuged at 7800 rpm for 5 min. The supernatant was discarded, and the resulting pellet of AuNC@GSH was dried under ambient airflow. The dried nanoclusters were then redispersed in DI by ultrasonication.

### Data Set Acquisition Method

The data set used in this work was compiled from peer-reviewed scientific publications reporting the synthesis of AuNCs stabilized by thiol-containing ligands. These studies provided detailed information about the synthesis conditions as well as corresponding fluorescence emission. In total, a data set of 207 experimental samples was used, each representing a unique synthesis condition. Data sets include the following independent variables: particles size (nm), pH, temperature (°C), and reaction time (h) of synthesis; ligand (categorical); measuring solvent (categorical). The emission wavelength was included as a dependent variable. The resulting data set was saved in txt. and xlsx. format.

### Algorithm

The relationship between the reaction conditions and their properties represents a complex nonlinear model, which is why the XGBoost algorithm was chosen, as it combines multiple weak classifiers into a strong classifier.^30^ The data set was divided into a training set (80%) and a test set (20%), using random sampling. The hyperparameters of the XGBoost model were selected manually with a conservative configuration without extensive searching, given the size of the data. The main values used were as follows: (i) number of estimators: 800; (ii) learning rate: 0.1; (iii) Max._ depth: 10; (iv) eval_metric: “rmse”; (v) eearly_stopping_rounds: 1; (vi) colsample_bytree: 0.8.

### Supervised Learning

Within the model, Decision Tree Regressor was used to make continuous predictions. This model is based on a decision tree structure, where the internal nodes represent tests on features, the branches illustrate the outcomes of those tests, and the leaves indicate an output value, in this case, the maximum emission wavelength of the gold nanoclusters AuNCs.

### Categorical Transformation

A technique known as One-Hot Encoding was used to convert categorical variables,^49^ which do not have an inherent order, into a format that can be provided to machine learning algorithms. Categorical variables were converted into a new column in the data set. When the categorical variable was the type of ligand, we defined three categories Ligand GSH: A, Ligand Thiol (articles that do not specify the thiol used): B, and Ligand alkyl thiolate: C. Thus, One-Hot Encoding created three columns: (i) ligand A: 1 if the ligand is A, 0 otherwise; (ii) ligand B: 1 if the ligand is B, 0 otherwise; (iii) ligand C: 1 if the ligand is C, 0 otherwise. Also, when the categorical variable was the measurement solvent, (i) organic solvent: A: 1 if the solvent is A, 0 otherwise and inorganic solvent: B: 1 if the ligand is B, 0 otherwise. Previously, the solvent was classified as organic (ethanol, methanol, acetone, toluene, hexane, hexanol, DMSO, DCM, THF, or DMF) and inorganic (buffer or water).

### Concerning Discrete Variables

By applying One-Hot Encoding, discrete variables, such as the types of thiols and the measurement solvent (organic or inorganic), are converted into numerical representations that can be easily interpreted by machine learning models.^49^ This method does not assign an order among the categories, thus preventing the model from assuming undesirable relationships between them.

### Assumption of Unknown Variables

Fixed values were assumed for unknown variables in cases where articles do not specify them. The most frequent value in the data set was assigned; for example, the temperature was set at 21 °C, the pH was considered neutral, the synthesis time was fixed at 24 h, the excitation wavelength was set at 365 nm, and the particle size was assumed to be 2 nm. This approach was used to ensure a more effective training of the model.

### Algorithm Selection and Evaluation

In this study, several approaches were explored, including polynomial fitting, multiple linear regression, neural networks, and support vector machines (SVM). Once the algorithms were selected, they were evaluated using appropriate metrics such as accuracy, based on the absolute and percentage errors of the data, and the coefficient of determination (*R* ^2^). In this case, the XGBoost algorithm demonstrated better performance compared to other algorithms such as AdaBoost (Figures S3 and S4).

## Supplementary Material



## Notes

Supporting figures and tables: dispersion analysis by variable, photography’s of AuNC@GSH dispersion, normal Q–Q plot of standardized residuals with AdaBoost, and tables containing the predicted and experimental values of the different tests. (PDF)

## Abbreviations

AuNC: gold nanoclusters
NIR: near-infrared
GXBoost: extreme gradient boosting
nm: nanometers
Q–Q: quantile–quantile
