# Supplementary material for: Predicting the Optical Properties of Gold Nanoclusters Using Machine Learning Approach
Source: ACS Omega. 2025 Oct 17;10(42):50200–7. doi: 10.1021/acsomega.5c06771 (PMC12573189; doi:10.1021/acsomega.5c06771)
Supplement: Supplementary file 1 [file ao5c06771_si_001.pdf]

Predicting the Optical Properties of Gold Nanoclusters Using Machine Learning Approach

Geraldine Sánchez-Dueñez[a] *Wladimiro Diaz-Villanueva,*[b] *Jorge Escorihuela,*[*a,c] *Laura Francés-Soriano*[*d] *and Julia Pérez-Prieto*[a]

[a] Institut de Ciència Molecular (ICMol), Universitat de València, C/ Catedrático José Beltrán 2, 46980, Paterna, Spain

[b] Institute for Integrative Systems Biology (I2SysBio), Universitat de València/CSIC, C/ Catedràtic Agustín Escardino Benlloch, 46980, Valencia, Spain.

[c] Departamento de Química Orgánica, Facultad de Farmacia y Ciencias de la Alimentación, Universitat de València, Avda. Vicente Andrés Estellés s/n, 46100, Burjassot, Spain.

[d] Grupo de procesos de oxidación avanzada, Departamento de Ingeniería Textil y Papelera, Universitat Politècnica de València, Campus d'Alcoi, 03801, Alcoi, Spain.

INDEX

Figure S1. Correlation between synthesis parameters and emission length in AuNCs: dispersion analysis by variable. 3

Figure S2. Photography's of AuNC@GSH in water under ambiental and UV light....3

Figure S3. Normal Q-Q plot of standardized residuals with AdaBoost. 4

Figure S4. Predicted *vs.* experimental emission wavelengths for training, testing and validation sets. 4

Table S1. Comparative experimental and predicted values in training set with thiols ligands data. 5

Table S2. Comparative experimental and predicted values in test set with thiols ligands data. 5

Table S3. Comparative experimental and predicted values in training set. 5

Table S4. Comparative experimental and predicted values in validation set. 10

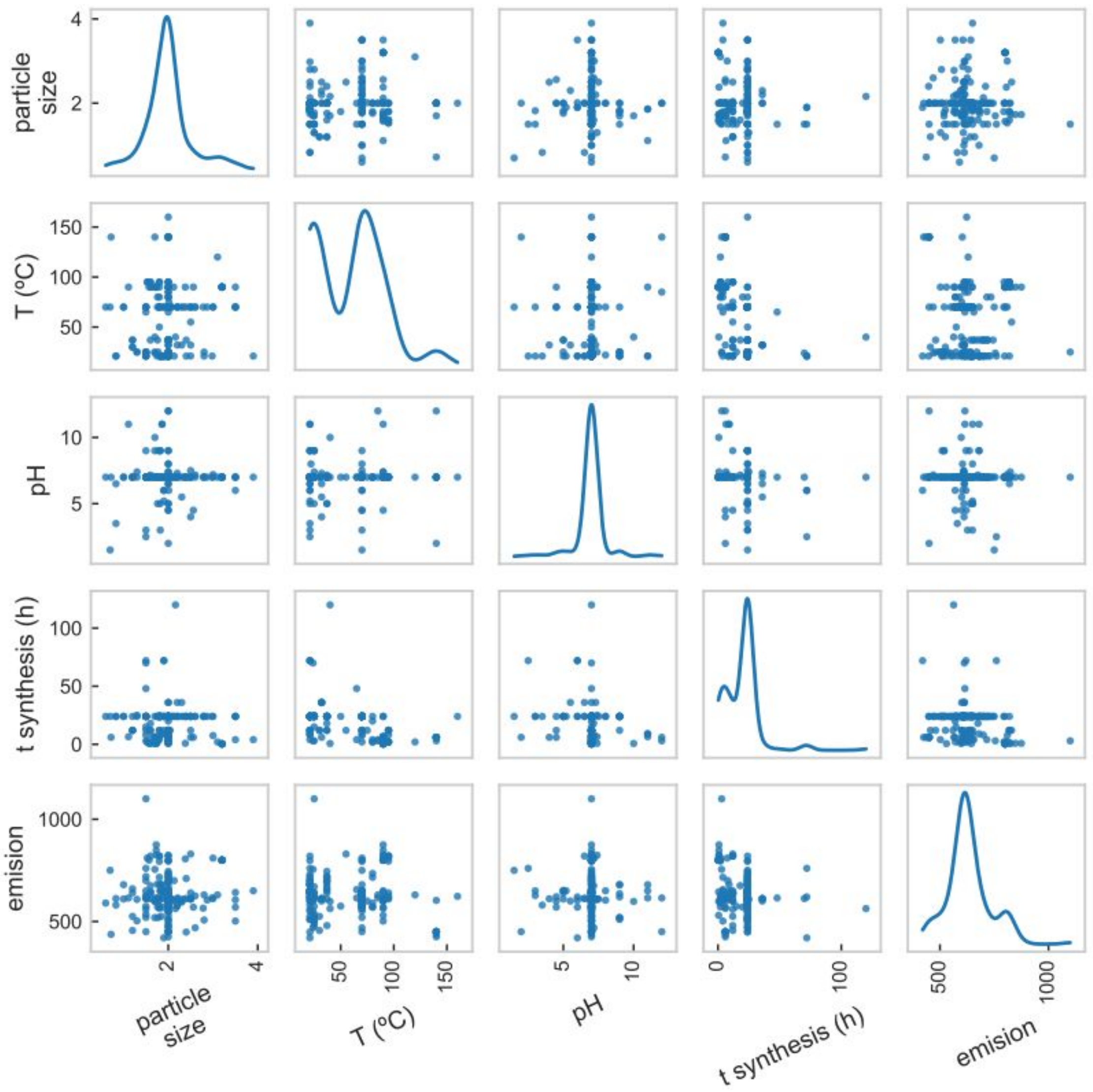

Figure S1. Correlation between synthesis parameters and emission length in AuNCs: dispersion analysis by variable.

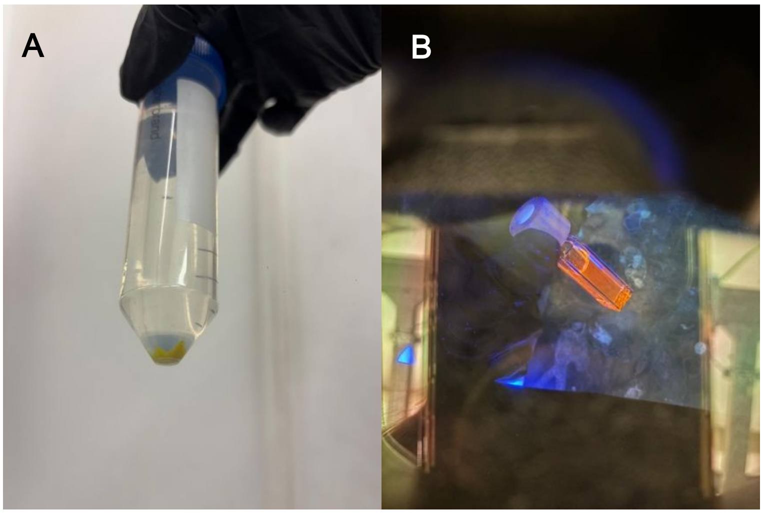

Figure S2. Photography's of AuNC@GSH in water under ambiental (A) and UV light (B).

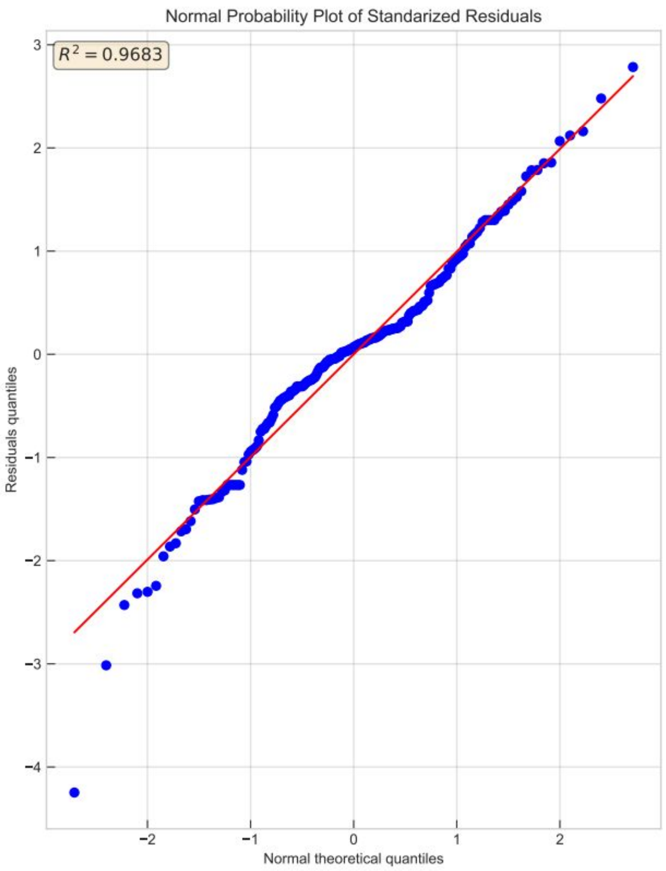

Figure S3. Normal Q-Q plot of standardized residuals with AdaBoost. Residuals (blue dot) compared to expected normal distribution (red line).

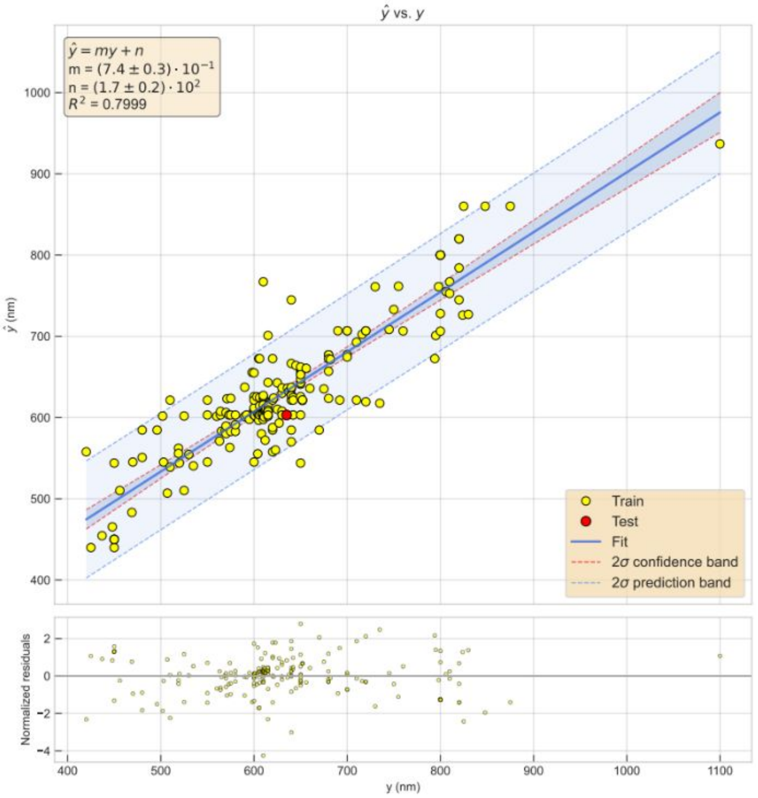

Figure S4. Predicted *vs.* experimental emission wavelengths for training, testing and validation sets.

Table S1. Comparative experimental and predicted values in training set with thiols ligands data.

Training Set			
**Entry**	**Experimental**	**Predicted**	**Relative error %**
1	448	448	0.00%
2	690	707.5	-2.54%
3	385	385	0.00%
4	720	707.5	1.74%
5	720	707.5	1.74%
6	371	371	0.00%
7	502	502	0.00%
8	735	735	0.00%
9	745	745	0.00%
10	700	707.5	-1.07%

Table S2. Comparative experimental and predicted values in test set with thiols ligands data.

Test Set			
**Entry**	**Experimental**	**Predicted**	**Relative error %**
11	700	643	8.14%
12	455	445.9	2.00%
13	700	707.5	-1.07%

Table S3. Comparative experimental and predicted values in training set.

Training Set			
**Entry**	**Experimental**	**Predicted**	**Relative error %**
1	810	716.7	11.5
2	571	613.6	-7.5
3	642	631.3	1.7
4	604	614.9	-1.8
5	450	609.5	-35.4
6	800	712	11.0
7	680	644.8	5.2
8	600	624.5	-4.1
9	700	668.2	4.5
10	630	629.4	0.1
11	1100	761.3	30.8
12	642	624.2	2.8
13	600	623.6	-3.9
14	420	595.4	-41.8
15	615	624.5	-1.5
16	598	623.9	-4.3
17	650	609.5	6.2

18	608	624.5	-2.7
19	794	635.4	20.0
20	610	624.5	-2.4
21	600	623.4	-3.9
22	604	623.4	-3.2
23	580	624.5	-7.7
24	800	703.4	12.1
25	600	630.5	-5.1
26	600	624.5	-4.1
27	575	624.5	-8.6
28	700	668.2	4.5
29	640	648.7	-1.4
30	425	542.9	-27.7
31	680	646.7	4.9
32	519	580.8	-11.9
33	595	624.5	-5.0
34	800	712	11.0
35	610	624.5	-2.4
36	800	660.4	17.5
37	825	735.4	10.9
38	810	666.5	17.7
39	612	628.3	-2.7
40	642	624.5	2.7
41	610	626.2	-2.7
42	710	640.5	9.8
43	590	619.5	-5.0
44	800	712	11.0
45	635	624.5	1.7
46	456	550.8	-20.8
47	680	646.7	4.9
48	745	666.4	10.6
49	450	542.9	-20.6
50	600	631.3	-5.2
51	530	621.5	-17.3
52	640	612.6	4.3
53	612	624.5	-2.0
54	450	547.2	-21.6
55	640	641.8	-0.3
56	606	635.4	-4.9
57	510	631.3	-23.8
58	606	635.4	-4.9
59	830	713.8	14.0
60	630	632.3	-0.4
61	610	644.8	-5.7
62	615	623.1	-1.3
63	610	624.5	-2.4

64	550	611.2	-11.1
65	480	612.6	-27.6
66	610	614.5	-0.7
67	645	632.2	2.0
68	592	624.5	-5.5
69	680	645.2	5.1
70	575	624.5	-8.6
71	610	624.5	-2.4
72	630	624.5	0.9
73	640	633.6	1.0
74	606	624.5	-3.1
75	800	712	11.0
76	600	611.2	-1.9
77	615	625.7	-1.7
78	625	617.9	1.1
79	600	626.2	-4.4
80	735	663.8	9.7
81	610	666.5	-9.3
82	550	631.3	-14.8
83	580	624.5	-7.7
84	635.9	631.2	0.7
85	570	609.1	-6.9
86	700	668.8	4.5
87	630	624.1	0.9
88	650	631.2	2.9
89	612	634	-3.6
90	720	660.6	8.3
91	570	611.1	-7.2
92	700	650.4	7.1
93	650	638.7	1.7
94	610	617.9	-1.3
95	800	712	11.0
96	750	674.8	10.0
97	610	620.5	-1.7
98	600	624.9	-4.2
99	760	689.5	9.3
100	469	586.4	-25.0
101	650	639.9	1.6
102	625	644.8	-3.2
103	610	630.9	-3.4
104	535	657.2	-22.8
105	606	624.2	-3.0
106	795	660.2	17.0
107	675	646.2	4.3
108	620	631.3	-1.8
109	565	617.9	-9.4

110	640	631.3	1.4
111	652	631.3	3.2
112	650	634.5	2.4
113	682	653.9	4.1
114	660	643.9	2.4
115	710	631.3	11.1
116	610	624.9	-2.4
117	720	668.2	7.2
118	612	624.2	-2.0
119	640	621	3.0
120	621	623.4	-0.4
121	615	624.5	-1.5
122	690	668.2	3.2
123	730	663.9	9.1
124	620	635.4	-2.5
125	615	660.2	-7.3
126	623	629	-1.0
127	670	612.6	8.6
128	612	617.9	-1.0
129	875	735.4	16.0
130	496	612.6	-23.5
131	575	618.7	-7.6
132	800	712	11.0
133	716	643.1	10.2
134	605	624.2	-3.2
135	610	624.5	-2.4
136	620	612.6	1.2
137	448	567.5	-26.7
138	507	589.2	-16.2
139	620	595.4	4.0
140	637.3	624.5	2.0
141	603	619.7	-2.8
142	820	677.3	17.4
143	503	575.8	-14.5
144	824	703.3	14.6
145	650	645.2	0.7
146	806	714.8	11.3
147	820	715.9	12.7
148	652	635.4	2.5
149	650	637.4	1.9
150	550	624.5	-13.5
151	656	632.2	3.6
152	650	624.5	3.9
153	437	541.9	-24.0
154	580	614	-5.9
155	627	621	1.0

156	480	598	-24.6
157	680	642.6	5.5
158	502	578.9	-15.3
159	574	618.9	-7.8
160	450	547.2	-21.6
161	611	625.8	-2.4
162	615	629.4	-2.3
163	605	635.4	-5.0
164	450	548	-21.8

Table S4. Comparative experimental and predicted values in validation set.

Validation Set			
**Entry**	**Experimetnal**	**Predicted**	**Relative error %**
165	615	624.9	-1.6
166	470	611.2	-30.0
167	640	624.5	2.4
168	820	648.7	20.9
169	600	687.8	-14.6
170	564	659.1	-16.9
171	615	647.1	-5.2
172	640	674.5	-5.4
173	755	705.9	6.5
174	608	572.9	5.8
175	570	632.9	-11.0
176	560	673.5	-20.3
177	525	550.8	-4.9
178	601	624.5	-3.9
179	520	609.5	-17.2
180	650	637.4	1.9
181	600	624.5	-4.1
182	613	626.2	-2.2
183	692	631.3	8.8
184	650	630.5	3.0
185	510	635.2	-24.5
186	820	715.9	12.7
187	573	624.5	-9.0
188	620	572.9	7.6
189	611	614.8	-0.6
190	600	663.2	-10.5
191	569	635.4	-11.7
192	848	735.4	13.3
193	652	628.7	3.6
194	564	624.5	-10.7
195	519	619.9	-19.4
196	563	612.5	-8.8
197	640	613.7	4.1
198	525	642.6	-22.4
199	610	620.6	-1.7
200	605	624.5	-3.2
201	610	664.5	-8.9
202	798	663.9	16.8
203	590	636.8	-7.9
204	720	668.2	7.2
205	450	547.2	-21.6
